# Supplementary material for: Cleavage of mitochondrial homeostasis regulator PGAM5 by the intramembrane protease PARL is governed by transmembrane helix dynamics and oligomeric state
Source: J Biol Chem. 2022 Jul 31;298(9):102321. doi: 10.1016/j.jbc.2022.102321 (PMC9436811; doi:10.1016/j.jbc.2022.102321)
Supplement: Supporting information [file mmc1.pdf]

Supporting information:

Cleavage of mitochondrial homeostasis regulator PGAM5 by the intramembrane protease PARL is governed by transmembrane helix dynamics and oligomeric state

Verena Siebert[1,2], Mara Silber[3], Elena Heuten[1,2], Claudia Muhle-Goll[3], Marius K. Lemberg[1,2,*]

[1]Center for Molecular Biology of Heidelberg University (ZMBH), Im Neuenheimer Feld 282, 69120 Heidelberg, Germany.
[2]Center for Biochemistry and Cologne Excellence Cluster on Cellular Stress Responses in Aging-Associated Diseases (CECAD), Medical Faculty, University of Cologne, Joseph-Stelzmann-Strasse 52, 50931 Cologne, Germany.
[3]Karlsruhe Institute of Technology (KIT), Fritz-Haber-Weg 6, 76131 Karlsruhe, Germany.
*Correspondence: m.lemberg@uni-koeln.de

Supporting figure legends

Figure S1. Bulky residue in P1 position shows only modest influence on PGAM5 processing.
(A) TM domain (TMD) and cleavage site region (marked by dotted line) of so far identified PARL substrates (17,28,29,87,95,96). **(B)** PGAM5 processing analyzed in cell-based PARL gain- and loss-of-function assay as in Fig. 1B. Ectopic expression of PARL wt but not the catalytic-inactive PARL^{S277A}, leads to increased processing. PGAM5 cleavage was stimulated by treating cells with the mitochondrial uncoupling agent CCCP. Grey arrowhead: 28 kDa cleavage fragment. Lower panel shows quantification of PGAM5 32/28 kDa distribution (n = 3, means ± SEM). Significant changes comparing PARL and PARL^{S277A} overexpression without and with CCCP treatment are indicated with black stars (*p ≤ 0.05, **p ≤ 0.01; unpaired two-tailed t-test). **(C)** Immunofluorescence analysis examines mitochondrial targeting of ectopically expressed PGAM5-FLAG constructs (purple) co-stained with endogenous TOM20 (green); cell nuclei are stained with Hoechst (blue). Scale bar, 5 µm. **(D)** For *OMA1* knockdown**,** cells were transiently transfected with either a non-targeting siRNA or an *OMA1*-specific siRNA for 48 hours before transient transfection of the PGAM5-FLAG contructs. PGAM5 cleavage was stimulated by treating cells with CCCP. Grey arrowhead: 28 kDa cleavage fragment. Lower panels each show quantification of PGAM5 32/28 kDa distribution (n = 3, means ± SEM). Significant changes versus cells transfected with non-targeting siRNA are indicated with black stars (*p ≤ 0.05, **p ≤ 0.01; unpaired two-tailed t-test).

Figure S2. PARL-catalyzed cleavage of PGAM5 is influenced by multiple TM residues.
(A) Western blot analysis of PGAM5 processing in a cell-based PARL gain- and loss-of-function assay as in Fig. 1B. Grey arrowhead: 28 kDa cleavage fragment. See Fig. 2B for quantification. **(B)** Immunofluorescence analysis examines mitochondrial targeting of ectopically expressed mutant PGAM5-FLAG constructs (purple) co-stained with endogenous TOM20 (green); cell nuclei are stained with Hoechst (blue). Scale bar, 5 µm. **(C)** Direct comparison of PGAM5 wt, S18L and S24F cleavage at endogenous PARL level without and with CCCP treatment. **(D)** Incubation of detergent-solubilized and purified recombinant PARL with MBP-PGAM5 leads to generation of an N-terminal cleavage fragment (NTF) as resolved by SDS-PAGE and staining with Coomassie blue. PARL-dependent alternative cleavage fragments appeared as side-effects of the detergent background. FL: MBP-PGAM5 full length. See Fig. 2C for quantification.

Figure S3. N-terminal substrate feature in PGAM5 important for PARL-catalyzed cleavage.
(A) Immunofluorescence analysis examines mitochondrial targeting of ectopically expressed mutant PGAM5-FLAG constructs (purple) co-stained with endogenous TOM20 (green); cell nuclei are stained with Hoechst (blue). Scale bar, 5 µm. **(B)** Direct comparison of PGAM5 wt, and C12S cleavage at endogenous PARL level without and with CCCP treatment. **(C)** *OMA1* levels are knocked down by transient transfection of *OMA1*-specific siRNA. Normalization of relative gene expression by comparison to the reference genes β-Actin (ACTB) and TATA-box binding protein (TBP). **(D)** For *OMA1* knockdown, cells were transiently transfected with either a non-targeting siRNA or an *OMA1*-specific siRNA for 48 hours before transient transfection of the PGAM5-FLAG contructs. PGAM5 cleavage was stimulated by treating cells with CCCP. Grey arrowhead: 28 kDa cleavage fragment. Right panel shows quantification of PGAM5 32/28 kDa distribution (n = 3, means ± SEM). No significant changes versus cells transfected with non-targeting siRNA were observed (unpaired two-tailed t-test). **(E)** Incubation of recombinant PARL with MBP-PGAM5^{C12S} leads to more efficient generation of the N-terminal cleavage fragment when compared with the wt construct (n = 3, means ± SEM; see Fig. 1C for comparison). PARL-dependent alternative cleavage fragments appeared as side-effects of the detergent background. FL: MBP-PGAM5 full length.

Figure S4. Structural properties of the PGAM5 TM domain.
(A) CD results of wt and three mutants. Values are scaled to wt values. **(B)** Hydrogen-deuterium exchange of wt TM domain shows stable H-bonds directly before the cleavage site

and at the N-terminus between Q8 an C12. Hydrogen bonds at the helix termini and between G13 and G17 are significantly weakened. Black dots indicate fast exchange, grey dots slow exchange. Some exchange rates could not be determined due to peak overlap, these residues are marked by an asterisk.

Figure S5. Negative charges in the PGAM5 juxtamembrane region influence cleavage efficiency under CCCP.
(A) Negatively charged cluster in juxtamembrane region of PINK1 but not PGAM5. Net charge in juxtamembrane region of negatively charged amino acids (black) and positively charged amino acids (grey). **(B)** Direct comparison of PGAM5 wt, and GG34/35EE cleavage at endogenous PARL level without and with CCCP treatment. **(C)** Immunofluorescence analysis examines mitochondrial targeting of ectopically expressed mutant PGAM5-FLAG constructs (purple) co-stained with endogenous TOM20 (green); cell nuclei are stained with Hoechst (blue). Scale bar, 5 µm. **(D)** For *OMA1* knockdown, cells were transiently transfected with either a non-targeting siRNA or an *OMA1*-specific siRNA for 48 hours before transient transfection of the PGAM5-FLAG contructs. PGAM5 cleavage was stimulated by treating cells with CCCP. Grey arrowhead: 28 kDa cleavage fragment. Below the quantification of PGAM5 32/28 kDa distribution (n = 3, means ± SEM). No significant changes versus cells transfected with non-targeting siRNA were observed (unpaired two-tailed t-test). **(E)** Putative disulfide (S-S) bond formation of PGAM5 wt and C12S samples was tested in reducing (red.) or non-reducing (non-red.) sample buffer conditions.

Figure S6. Formation of the PGAM5 higher order structure prevents PARL-catalyzed cleavage.
(A) Immunofluorescence analysis examines mitochondrial targeting of ectopically expressed oligomerization-defecient mutant PGAM5-FLAG constructs (purple) co-stained with endogenous TOM20 (green); cell nuclei are stained with Hoechst (blue). Scale bar, 5 µm. **(B)** Direct comparison of PGAM5 wt, ΔC and AAxxAA cleavage at endogenous PARL level without and with CCCP treatment. **(C)** For *OMA1* knockdown, cells were transiently transfected with either a non-targeting siRNA or an *OMA1*-specific siRNA for 48 hours before transient transfection of the PGAM5-FLAG contructs. PGAM5 cleavage was stimulated by treating cells with CCCP. Grey arrowhead: 28 kDa cleavage fragment. Lower panels show quantification of PGAM5 32/28 kDa distribution (n = 3, means ± SEM). No significant changes versus cells transfected with non-targeting siRNA were observed (unpaired two-tailed t-test). **(D)** Illustration of different detection levels between MG132 (2 µM, 24 h) treated and untreated samples of PGAM5 wt and ΔC when loaded beside each other. Due to in general lower protein levels of monomeric $PGAM5^{\Delta C}$, no protein could be detected when loaded beside the MG132-stabilized samples. **(E)** Analysis of monomeric PGAM5 double mutants additionally containing TM domain mutations G17L and S18L ($PGAM5^{\Delta C/G17L}$, $PGAM5^{\Delta C/S18L}$). Grey arrowhead: 28 kDa cleavage fragment. Right panel shows quantification of PGAM5 32/28 kDa distribution (n = 3, means ± SEM). Significant changes versus wt PGAM5-FLAG are indicated with black stars, significant changes versus $PGAM5^{\Delta C}$-FLAG are indicated with grey stars (*p ≤ 0.05, **p ≤ 0.01, ***p ≤ 0.001; unpaired two-tailed t-test).

Figure S7. Hypothetical model of the PGAM5 TM domain bound by a putative PARL exosite.
Model of PARL generated by AlphaFold, entry Q9H300. Catalytic S277 and H335 are depicted in red facing the water-filled cavity of PARL, which opens to the matrix. Insertion depth for PARL AlphaFold model and the helical PGAM5 TM domain examinded by NMR into the inner mitochondrial membrane was determined with the OPM server. The amphipathic helix of PGAM5 is shown here with a submerged orientation that allows the charged and hydrophilic sidechains to be placed within the lipid headgroup area. Cleavage site within the C-terminal helix of PGAM5 TM domain (F23-S24) is depicted in magenta. Red area: upper lipid layer towards mitochondrial IMS, blue area: lower lipid layer towards mitochondrial matrix.

Siebert *et al.* - Figure S1

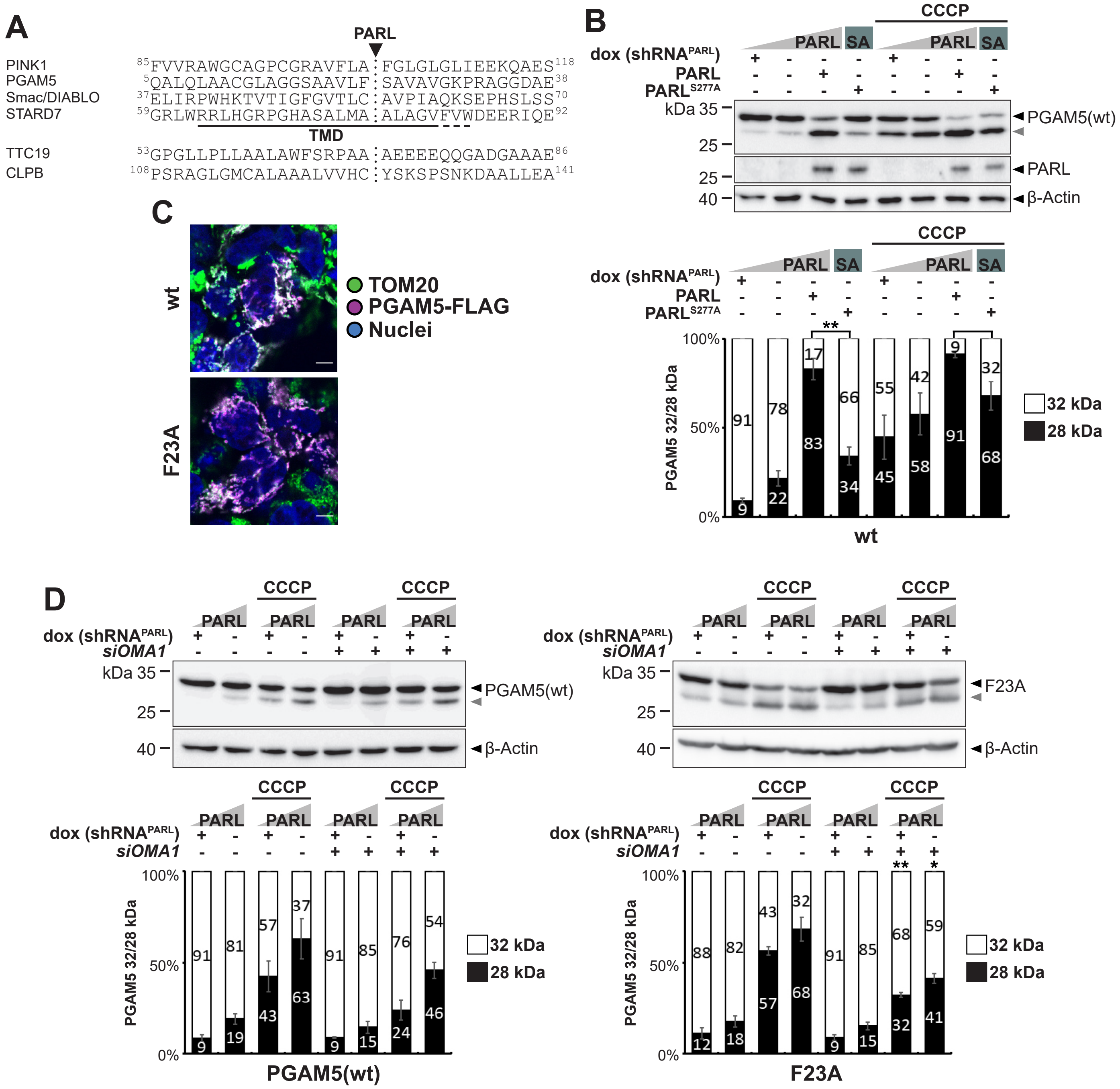

Siebert *et al.* - Figure S2

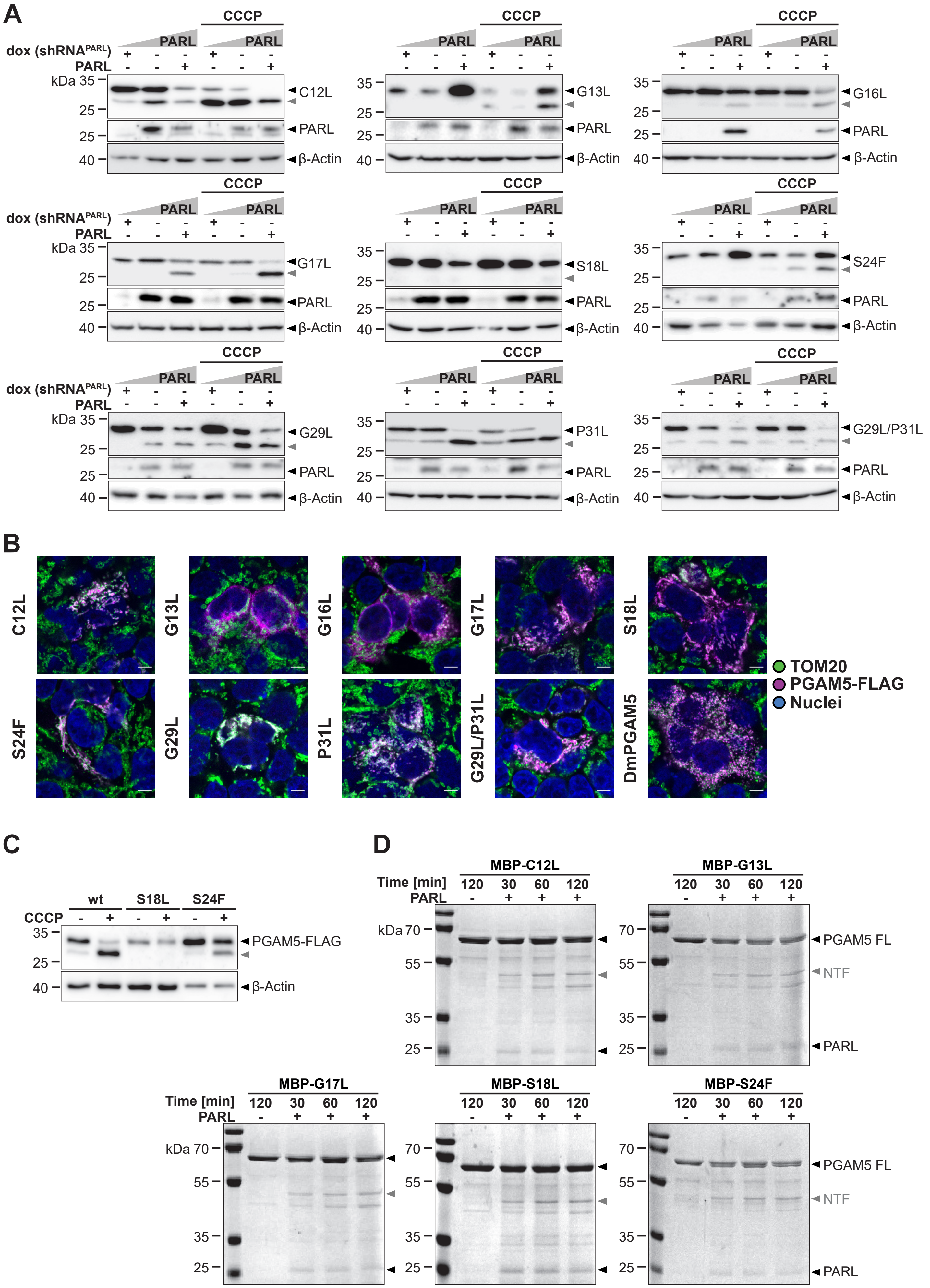

Siebert *et al.* - Figure S3

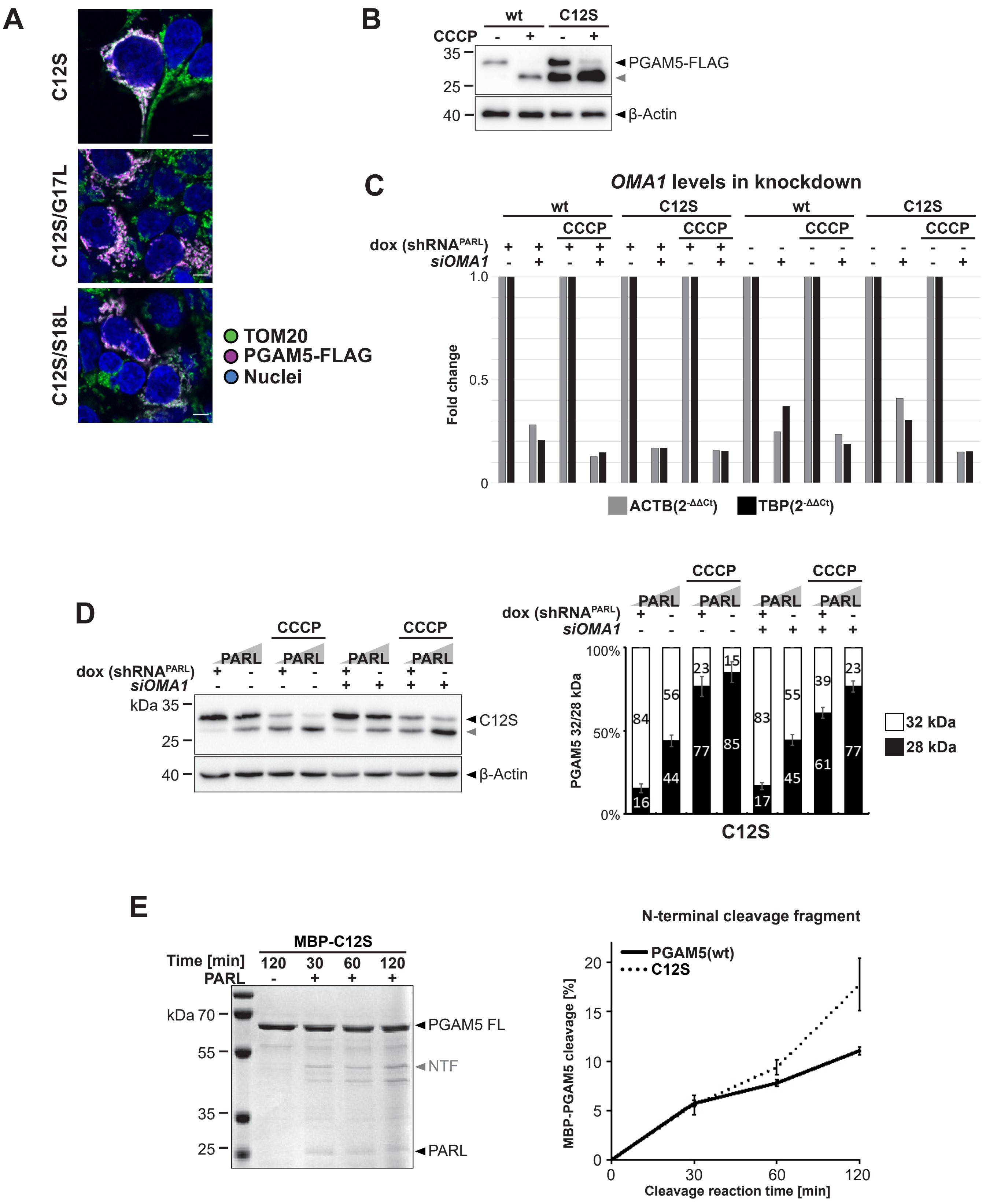

Siebert *et al.* - Figure S4

A

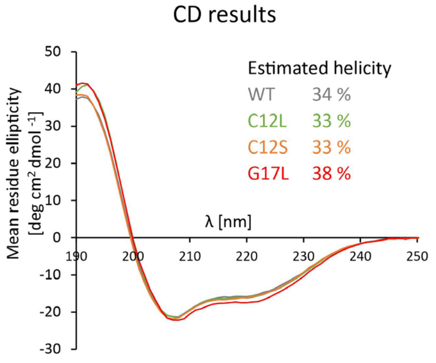

B

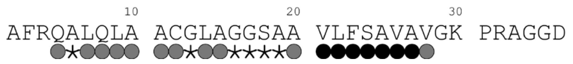

Siebert *et al.* - Figure S5

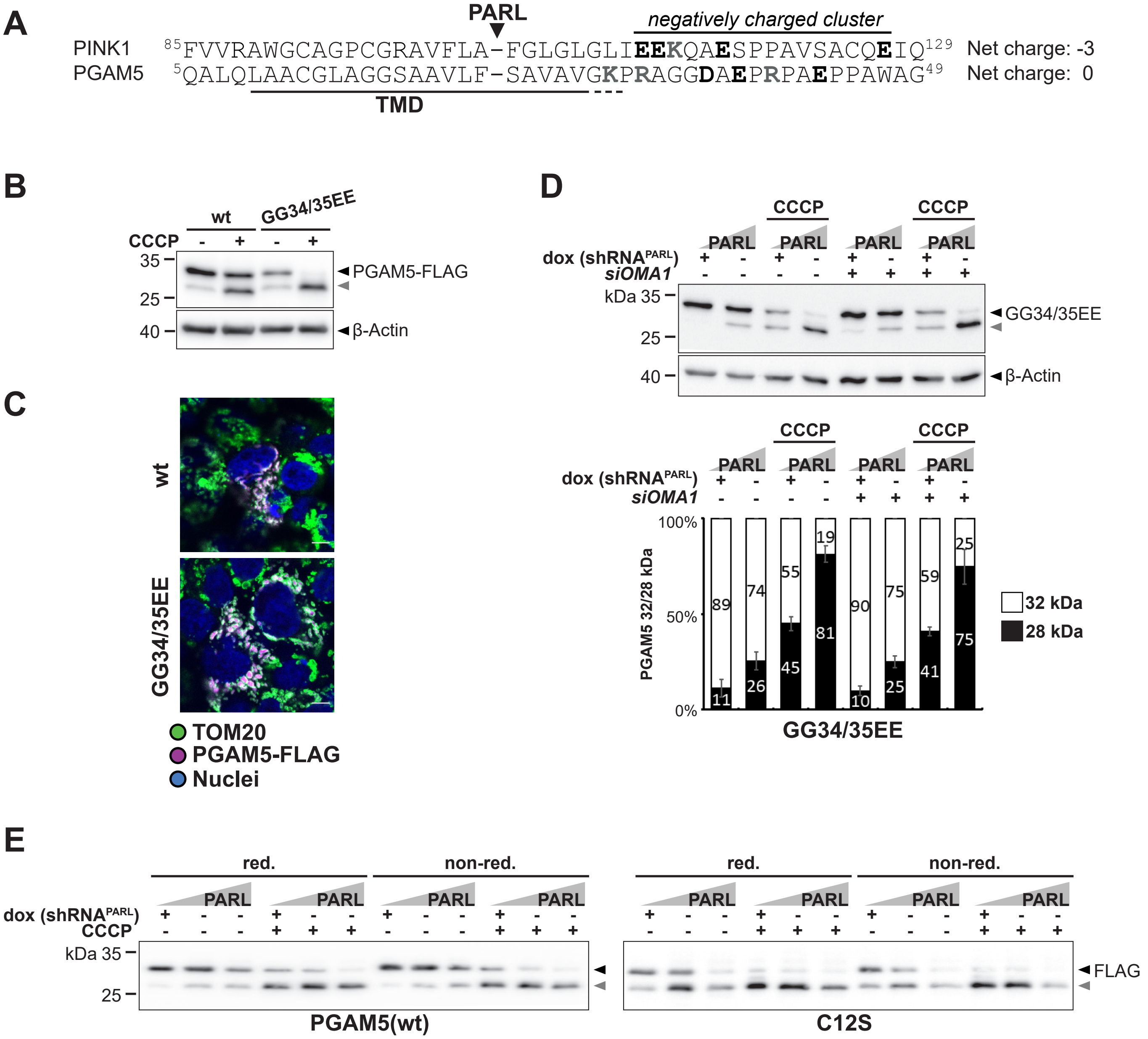

Siebert *et al.* - Figure S6

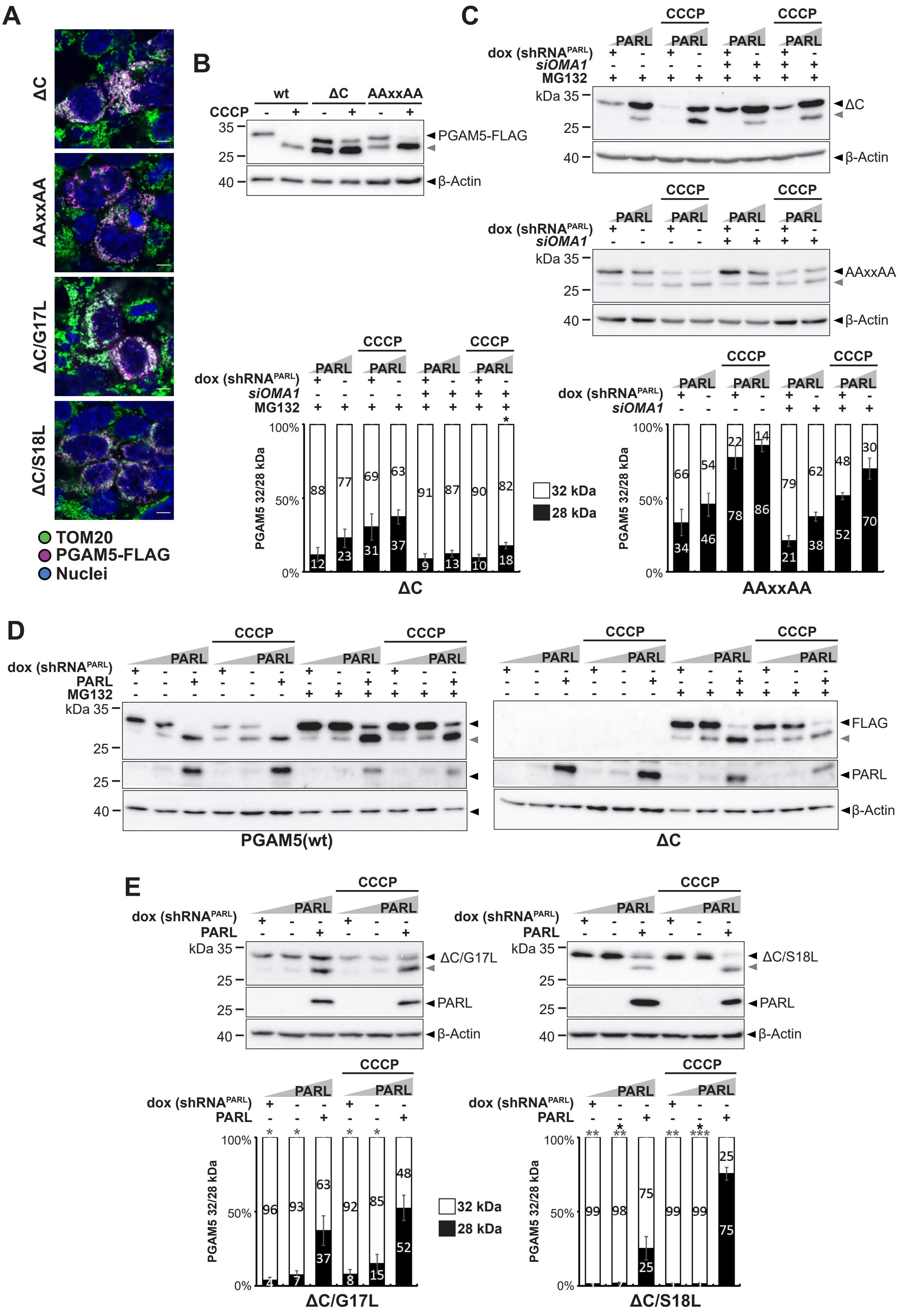

Siebert *et al.* - Figure S7

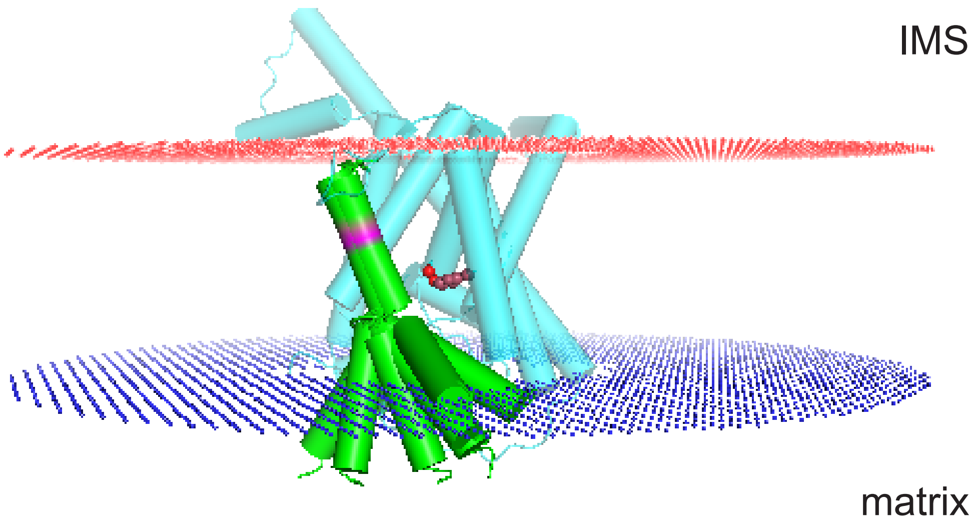

Table S1. Structure statistics of PGAM5 WT TMD and four single point mutants.

	WT	C12L	C12S	G17L
**Total restraints used**	269	279	261	274
unambiguous NOE restraints	269	279	261	274
Intraresidue	106	107	124	127
Sequential ($\lvert i-j \rvert=1$)	85	87	87	93
Medium range ($1 < \lvert i-j \rvert < 4$)	61	66	47	40
Long range ($\lvert i-j \rvert \geq 4$)	17	19	3	17
Ambiguous NOE restraints	0	0	0	0
**Statistics for structure calculations**				
RMSD of bonds (A)	0.001+/- 0.00007	0.001 +/- 0.00007	0.001 +/- 0.00006	0.001 +/- 0.0001
RMSD of bond angles (°)	0.253 +/- 0.005	0.260 +/- 0.005	0.252 +/- 0.004	0.285 +/- 0.011
RMSD of improper torsions (°)	0.108 +/- 0.007	0.098 +/- 0.007	0.102 +/- 0.007	0.146 +/- 0.02
**Final Energies (kcal mol^{-1})**				
E_{total}	-1044 +/- 30	1057 +/- 24	-1034 +/- 30	-1058 +/- 22
E_{bonds}	0.369 +/- 0.064	0.360 +/- 0.059	0.403 +/- 0.056	0.566 +/- 0.121
E_{angles}	8.35 +/- 0.33	8.98 +/- 0.32	8.24 +/- 0.26	10.90 +/- 0.89
$E_{impropers}$	0.432 +/- 0.057	0.362 +/- 0.055	0.387 +/- 0.053	0.823 +/- 0.287
E_{dihed}	134.0 +/- 0.98	134.2 +/- 0.81	133.3 +/- 0.82	140.2 +/- 1.78
E_{vdW}	-207.6 +/- 4.3	-203.22 +/- 4.28	-195.96 +/- 3.2	-210.1 +/- 2.7
E_{NOE}	-980.1 +/- 28.7	-988.25 +/- 25.5	-981.0 +/- 29.5	-1001 +/- 22
**Coordinate precision (A)**				
RMSD of backbone (N,CA,C,O) of all residues	3.06	4.47	4.20	4.35
RMSD of all heavy atoms of all residues	3.56	5.11	5.05	5.05
RMSD of backbone (N,CA,C,O) of ordered residues (3:28)	2.18	2.30	2.95	3.42
RMSD of all heavy atoms of ordered residues (3:28)	2.67	2.96	4.0	2.37

All values refer to the ensemble of 20 structures with the lowest energy from 400 calculated structures. NOE: Nuclear Overhauser Effect, RMSD: root-mean-square deviation of atomic positions.
